# Comment on “Efficient full-path optical calculation of scalar and vector diffraction using the Bluestein method”

**DOI:** 10.1038/s41377-020-00447-9

**Published:** 2021-01-11

**Authors:** Yifeng Shao, H. Paul Urbach

**Affiliations:** Optics Research Group, Imaging Physics Department, Faculty of Applied Sciences, Delft University of Technology, Lorentzweg 1, 2628 CJ Delft, The Netherlands

**Keywords:** Optics and photonics, Imaging and sensing

The calculation of light diffraction is of great importance for many essential optical applications, including optical lithography, optical tweezers, and super-resolution imaging, and is a research topic that has been extensively studied over the past few decades. Because both scalar and vector diffraction can be formulated as Fourier transforms, the standard method is to use the fast Fourier transform (FFT) algorithm. However, the use of the FFT requires a fixed sampling relation between the discretization of the input field and that of the output field. For the vectorial case, this requirement often causes a significant waste of computational resources, which hinders real-time applications.

Recently, Hu et al.^1^ proposed a method for calculating scalar and vector diffraction that is both efficient and flexible in choosing the sampling grid. The proposed method is based on the Bluestein method. In particular, the authors calculated the discrete Fourier transform (DFT) with input array length M and output array length N using the chirp *z*-transform (CZT) algorithm instead of the FFT algorithm. The Bluestein method is a crucial step in the development of the CZT algorithm, as it reformulates the DFT as a convolution and hence enables the efficient calculation of the DFT. For further details, please refer to Eq. (11) in the original paper of the CZT algorithm^2^.

The proposed method is, however, not novel. In fact, an identical approach was proposed in 2006 by Leutenegger et al.^3^. The core formula, Eq. (12), in the paper by Hu et al.^1^ resembles Eq. (18) in ref. ^3^ except for an exchange of the indices M and N. The use of the CZT algorithm remains valid as long as the diffraction is described by the Fourier transform. Paper^3^ considers the calculation of a focus field with a high NA, while the discussions in the paper by Hu et al.^1^ do not go beyond this scope.

Note that the paper by Hu et al.^1^ does cite paper^3^ as reference 15 in the Introduction when mentioning the FFT algorithm: “Fast Fourier transform (FFT)-based algorithms have been developed to perform fast calculations of light diffraction [15,16,17,18,19]”. However, although the main content of paper^3^ (reference 15) is related to the CZT algorithm, this information is neglected in ref. ^1^.

Paper^3^ has been cited more than 150 times since its publication in 2006. Actually, calculating light diffraction using the CZT transform has become the new standard in many imaging and focusing models.

The authors claimed that the original article consists of three main parts: scalar diffraction, vector diffraction, and full-path propagation. Among these three parts, the scalar case is trivial, and the vector case has been excessively studied in the literature. We agree with the reviewers that the demonstrated calculation of the full-path propagation is new. However, the authors did not propose any novel method that enables this calculation. We doubt that the authors can develop a complete research article for publication in the *Light* journal based solely on the topic of full-path propagation.
